# Cooperative problem solving in giant otters (*Pteronura brasiliensis*) and Asian small-clawed otters (*Aonyx cinerea*)

**DOI:** 10.1007/s10071-017-1126-2

**Published:** 2017-08-24

**Authors:** Martin Schmelz, Shona Duguid, Manuel Bohn, Christoph J. Völter

**Affiliations:** 1 Department of Developmental and Comparative Psychology, Max Planck Institute for Evolutionary Anthropology, Leipzig, Germany; 2 Department of Cognitive Biology, University of Vienna, Vienna, Austria; 3 The Otter Project, Leipzig, Germany; 4 School of Psychology and Neuroscience, St Andrews University, St Andrews, UK

**Keywords:** Lutrinae, Giant otter, Asian small-clawed otter, Loose string task, Cooperation, Social cognition

## Abstract

Cooperative problem solving has gained a lot of attention over the past two decades, but the range of species studied is still small. This limits the possibility of understanding the evolution of the socio-cognitive underpinnings of cooperation. Lutrinae show significant variations in socio-ecology, but their cognitive abilities are not well studied. In the first experimental study of otter social cognition, we presented two species—giant otters and Asian small-clawed otters—with a cooperative problem-solving task. The loose string task requires two individuals to simultaneously pull on either end of a rope in order to access food. This task has been used with a larger number of species (for the most part primates and birds) and thus allows for wider cross-species comparison. We found no differences in performance between species. Both giant otters and Asian small-clawed otters were able to solve the task successfully when the coordination requirements were minimal. However, when the temporal coordination demands were increased, performance decreased either due to a lack of understanding of the role of a partner or due to difficulty inhibiting action. In conclusion, two species of otters show some ability to cooperate, quite similar to most other species presented with the same task. However, to draw further conclusions and more nuanced comparisons between the two otter species, further studies with varied methodologies will be necessary.

**Electronic supplementary material:**

The online version of this article (doi:10.1007/s10071-017-1126-2) contains supplementary material, which is available to authorized users.

## Introduction

Cooperation can take many forms in social carnivores, from cooperative hunting, to territory defense, intragroup alliances and cooperative breeding. The cognitive processes resulting in these various forms of cooperation are likely to be equally varied and have thus been the subject of much experimental research with captive populations. These studies investigate not only the conditions under which cooperation is successful but also what individuals understand about cooperation, for example, what they understand about the need for cooperative partners and how they use this understanding to solve problems cooperatively. The comparison of these cooperative abilities between species has important implications for understanding the evolution of cooperation and cognition (Brosnan et al. 2010; Burkart and van Schaik 2010; Byrne and Whiten 1988; Dunbar 2009).

A simple experimental paradigm, the “loose string task,” has been used to investigate cooperation in a variety of species, including primates (e.g., Hirata and Fuwa 2007; Melis et al. 2006a; Molesti and Majolo 2016), birds (e.g., Massen et al. 2015; Seed et al. 2008), and elephants (Plotnik et al. 2011), but no carnivores have been studied, except for domestic dogs (Ostojić and Clayton 2014), which are a special case because of the effects of human selection. This task focuses on mutualistic cooperation in which individuals need to coordinate their actions with others to gain rewards they would not be able to access individually. Originally designed for chimpanzees, the task requires two individuals to pull simultaneously on either end of a rope to pull in a food reward. The rope is set up so that if only one pulls, the other end of rope moves out of reach and the food remains inaccessible (Hirata and Fuwa 2007). Success in this task suggests an ability to coordinate actions with conspecifics. However, a stronger test of what individuals understand about the role of a partner for successful cooperation is the delayed version of the task (also part of the original design in Hirata and Fuwa 2007). In this case, initially only one subject is given access to the rope and they have to wait until a partner arrives before pulling. Importantly, this version rules out the possibility that success is achieved by individuals accidently pulling at the same time. In order to pass the delay task, subjects need to understand that a partner is necessary for success. Chimpanzees not only wait for a partner, but are able to recruit a partner when necessary (Melis et al. 2006a) and can also choose a competent partner over an incompetent one (Melis et al. 2006b). Few other species have been presented with this version of the task: rooks (Seed et al. 2008); ravens (Massen et al. 2015); African grey parrots (Péron et al. 2011); kea (Heaney et al. 2017); domestic dogs with human partners (Ostojić and Clayton 2014); and Asian elephants (Plotnik et al. 2011). Of these, only Asian elephants and kea were able to wait for a partner for an extended period of time (up to 45 s for elephants and 65 s for kea) and domestic dogs for a shorter period (2.2 s on average).

**Fig. 1:**
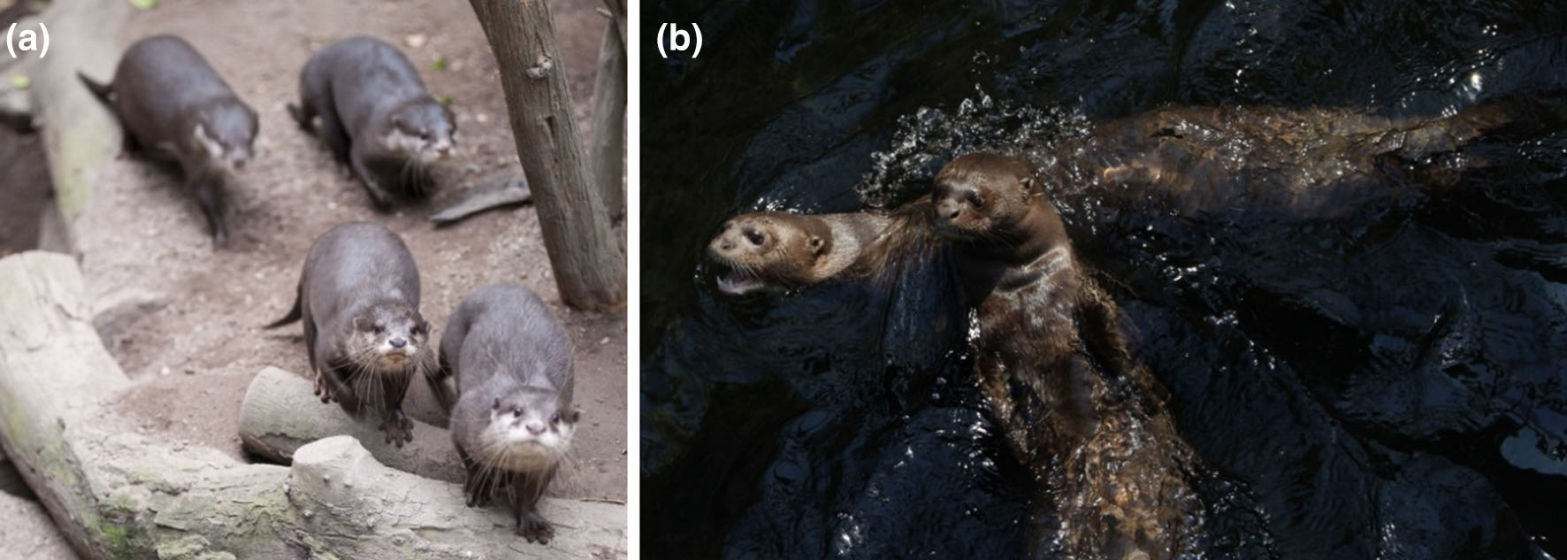
The two study species. **a** Asian small-clawed otters (photograph by Isabelle Grubert) and **b** giant otters (photograph by Shona Duguid) in Zoo Leipzig

In the current study, we compare two species of otter on the loose string task: giant otters (*Pteronura brasiliensis*) and Asian small-clawed otters (*Aonyx cinerea*; Fig. 1).

To date, very little is known about the cognitive abilities of otters (Lutrinae) and the experimental work that has been conducted is spread across species, particularly with regard to social cognition. Most notably, call-back experiments suggest that both giant and Asian small-clawed otters can recognize individual callers (Lemasson et al. 2013; Mumm et al. 2014) and small-clawed otters show evidence of spatial memory for food locations (Perdue et al. 2013).

Although the experimental research is sparse, more is known about the behavior of wild populations, at least for giant otters. Giant otters are the largest species of otter and are found mainly in Brazilian river systems (Kruuk 2006). They are cooperative breeders that live in groups of up to 20 (though usually around 3–9 individuals; Duplaix 1980; Groenendijk et al. 2014) consisting of a breeding pair, their young, and older helpers that babysit (Rosas et al. 2009) and provision the young (Kruuk 2006). As well as being cooperative breeders, groups will jointly defend their territory from predators such as caiman and forage together (Duplaix 1980), with some indication they hunt fish cooperatively (Staib 2002).

Asian small-clawed otters are the smallest otter species; they are found in wetland habitats across India, South East Asia and southern China (Hussain et al. 2011). They also live in social groups; however, much less is known about their socio-ecology as most observations of their social structure were made in captive populations (Hussain et al. 2011). They live in extended family groups of up to 15 individuals (Kruuk 2006), with both parents involved in upbringing of the young (Hussain et al. 2011). In contrast to giant otters, small-clawed otters generally forage individually (Kruuk et al. 1994).

Overall, both species are social but the evidence suggests that coordinated cooperative activities, particularly in foraging contexts, play a more important role in the lives of giant otters. Thus, when presented with a new cooperative problem-solving task that involves coordination, we expect giant otters to outperform Asian small-clawed otters. We presented captive otters, one group of giant otters (*N* = 5) and one group of Asian small-clawed otters (*N* = 4) with the simultaneous and delay versions of the loose string task to investigate their abilities to cooperate with each other. Following Massen et al. (2015) and Molesti and Majolo (2016), we tested both species in a group setting, with all group members present. This reflects the setting in which cooperative problems would be solved in the wild.

## Methods

### Subjects

Two family groups of otters—five giant otters (*Pteronura brasiliensis*) and four Asian small-clawed otters (*Aonyx cinerea*)—participated in this study. None of the subjects had previous experience with experimental studies. The animals were housed at Leipzig zoo. The giant otter group consisted of an adult female (9.5 years) with her four subadult offspring (2 females, all 1.5 years of age). The small-clawed otter group consisted of four males (siblings) aged between 5.0 and 6.6 years (see Supplementary Material 1 for details concerning husbandry and enclosures). One juvenile giant (Erna) otter stopped participating during the training and was therefore not included in any analysis.

### Materials

**Fig. 2:**
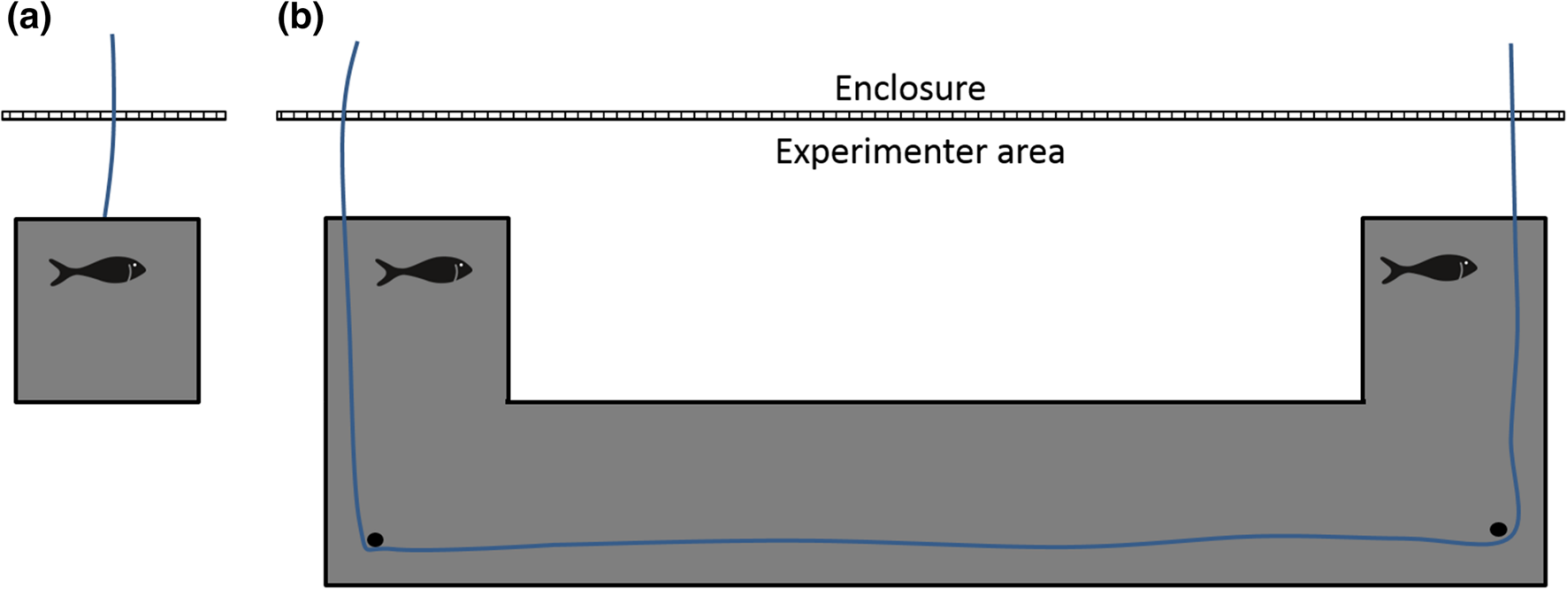
Illustrations of the two types of apparatus used in the current study. **a** Individual training, **b** cooperation test

The individual training apparatus consisted of small, square PVC platforms (giant otters: 30 × 30 cm, small-clawed otters: 15 × 15 cm) with a rope attached to it (see Fig. 2a). The cooperation apparatus was modeled on the original design by Hirata and Fuwa (2007). Our “Hirotter” board consisted of a long, flat, U-shaped platform (giant otters: 200 × 60 cm, small-clawed otter: 100 × 30 cm, see Fig. 2b). The training and test boards were located on the floor outside the enclosure. During the test, we baited both ends of the board with food rewards with preferred food types as indicated by the caretakers (pieces of fresh fish for the giant otters and cat food for the small-clawed otters, after trying grapes in the first sessions). A rope ran around three vertical screws (for the giant otters) or through two eyebolts (for the small-clawed otters) that were protruding from the platform at both ends (and in the middle for the giant otters). At both sides of the platform, the ends of the rope extended into the otter enclosure underneath the mesh. The otters could access the food on the platform if two individuals were cooperating either by pulling at each end of the rope simultaneously or by holding one end of the rope, while the partner was pulling the other end of the rope. One individual pulling the rope alone resulted in removal of the rope from the apparatus without moving the baited platform. Thus, pulling only one end of the rope resulted in loss of access to the food as the second individual could no longer reach the rope.

### Procedure

The entire study was conducted in a group setting per species, i.e., no individuals were separated from the group at any point. Subjects were first trained in an individual string pulling task before they entered the cooperation test phase (see Supplementary Material 1 for details). The cooperation test phase encompassed five conditions that were administered in this order: Simultaneous I (6 sessions/103 trials), Simultaneous II (6 sessions/93 trials), Delay I (3 sessions/28 trials), Long-rope-delay (3 sessions/29 trials) and Delay II (1 session/14 trials) (see Supplementary Material 2 for examples of simultaneous and delay trials in both species). For the giant otters, the number of trials per session varied (between 5 and 30 trials) depending on the food availability as the amount and size of fish provided to us by the zoo varied. For the small-clawed otters, we matched the number of trials to the giant otters. In all conditions, both sides of the platform were baited at the same time. In the simultaneous conditions, the experimenters slid both ends of the rope underneath the mesh of the enclosure at the same time when at least one subject was present on each side of the apparatus. Subjects could therefore access the two ends of the rope simultaneously; no waiting was necessary. When one individual pulled harder than the other one, the platform sometimes tilted so that one side of the platform became accessible before the other one. When this happened, the former individual typically released the rope to eat the food. For this reason, the other individual could not retrieve its food reward. In the Simultaneous I condition, this resulted in an uneven food distribution in some trials (proportion of trials with uneven food distribution in Simultaneous 1: giant otters: 0.40; small-clawed otters: 0.20). In Simultaneous II, the experimenters pushed the other side of the platform forward when the platform tilted to maintain a consistent reward contingency.

**Fig. 3:**
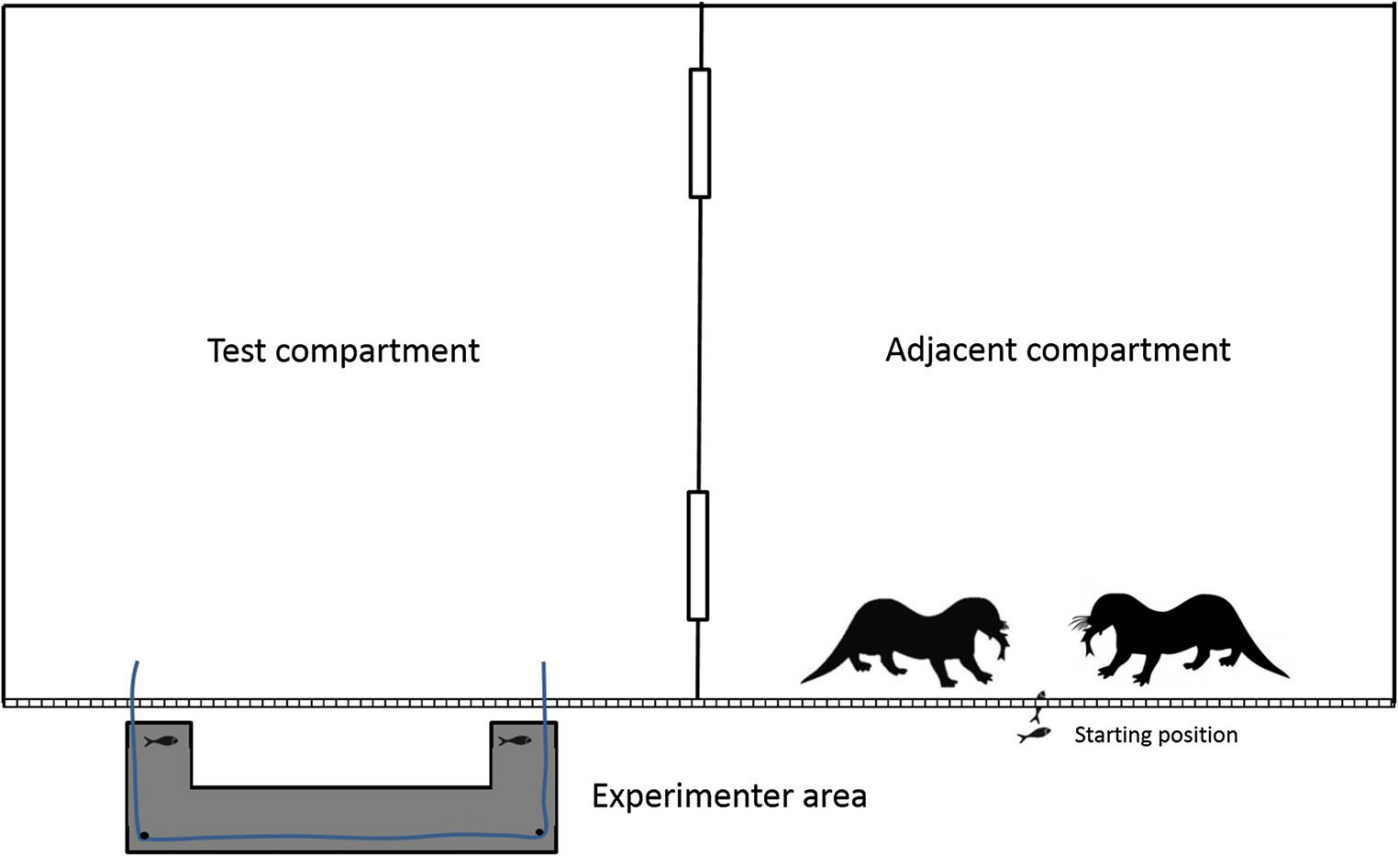
Illustration of the setup in the delay conditions. Subjects were lured to the adjacent compartment, while the Hirotter board was baited. One end of the rope was closer to the door to the adjacent compartment than the other one so that the returning subjects could access this end of the rope before the other one

In the delay conditions, all individuals in the group were lured to another compartment of the enclosure as far from the apparatus as possible where every individual would receive a piece of food. While the test compartment was empty, the platform was baited and the two ends of the rope were pushed into the test compartment (see Fig. 3). The delayed access to the rope ends was achieved by the delayed entry of the otters because one rope was closer to the door to the adjacent compartment, so that when the otters returned to the testing compartment, the first individual could access this end first and they would have to wait before another individual could move around to the other end of the rope. In Delay I and II, the rope was the same length as in the simultaneous conditions (giant otters: 4.0 m total length, approx. 0.3 m inside the cage at either end; small-clawed otters: 2.0 m, approx. 0.15 m inside). In the Long-rope-delay condition, we extended the length of the rope, thereby relaxing the need for temporal synchronization of pulling (giant otters: 5.4 m, approx. 1 m inside; small-clawed otters: 2.7 m, approx. 0.5 m inside) and providing the otters with further opportunity to learn the affordances of the delay conditions.

### Coding and analysis

For each trial, we coded whether or not the participating otters were successful. Trials in which the board was pulled in only on one side were also coded as success (proportion of all trials: giant otters: 0.45; small-clawed otters: 0.27). A second coder, blind to the purpose of the study, coded a random selection of 20% of test trials from video. There was a very high agreement of 96.36% between the two coders (Cohen’s Kappa; *Κ* = 0.92). Furthermore, we coded live which subject pulled on which end of the rope (left or right). In most unsuccessful trials, one subject started pulling on the rope, while the other end was unoccupied. For these trials, we coded the subject who pulled on the rope. For the delay conditions, a third coder coded the time between the arrival of the first otter and the arrival of the second otter at the board from video.

The dependent variable was the binary success code. To analyze the data, we used a generalized linear mixed model (GLMM) with a binomial error structure. All models were fitted in R (R Core Team 2012) using the function glmer of the R-package lme4 (Bates et al. 2015). We used likelihood ratio tests (LRT) to assess whether the inclusion of predictors and their interactions improved the general fit of a model to the data by comparing models with and without the respective effects (Dobson and Barnett 2008).

The full model comprised of species, condition and their interaction as fixed effects and trial and session number as covariates. We compared this model to a reduced model comprising of only the covariates (trial number and session number). To test the significance of the interaction, we compared the full model to a reduced model without the interaction. Given the interaction turned out to be nonsignificant, we tested the significance of each fixed effect (species and condition) by comparing a model comprising them to a model lacking them. We accounted for the identity of the first individual pulling one end of the rope (left and right) and the specific dyad by including them as random intercept terms (see Supplementary Material 1 for details).

## Results

**Fig. 4:**
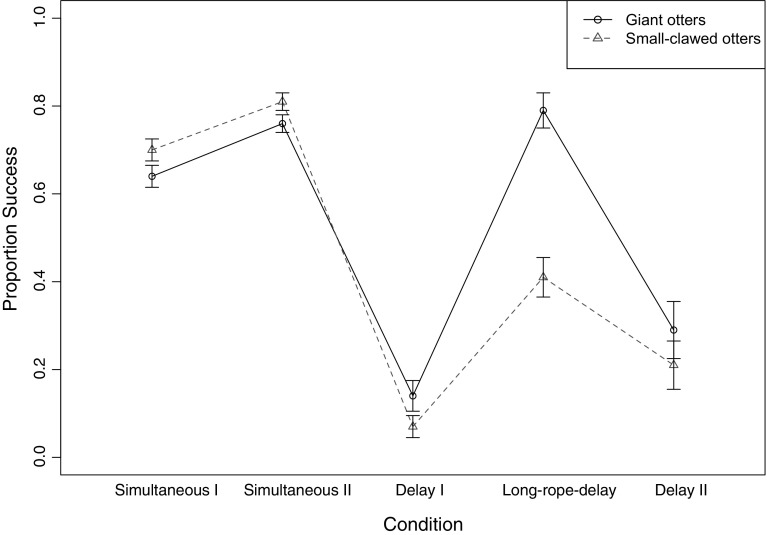
Proportion of successful trials per condition and otter species

The full model of coordination success comprising of species, condition and their interaction fit the data better compared to models lacking them [LRT: *χ* ^2^(9) = 39.05, *p* < .001]. The interaction term between species and condition did not improve the model fit [LRT: *χ* ^2^(9) = 2.13, *p* = .713]. In the final models without the interaction, we found a significant effect of condition [LRT: *χ* ^2^(4) = 34.61, *p* > .001] but no significant differences between species [LRT: *χ* ^2^(1) = 1.20, *p* = .283]. Figure 4 shows the proportion of successful trials for each condition in the two species; Table 1 shows the average estimates, *p* values and confidence intervals for the final model.

**Table 1:** Average parameters for the final model based on 1000 iterations

	*β*	SE (*β*)	*p*	95% CI	
				LL	UL
Intercept	−0.119	0.729			
Condition^a^					
Simultaneous II	0.755	0.386	.058^†^	−0.059	1.669
Delay I	−2.830	0.550	.001**	−10.293	−1.899
Long-rope-delay	−1.090	1.086	.318	−4.008	0.937
Delay II	−2.492	1.677	.115	−11.358	−0.607
Species^b^	0.777	0.740	.296	−0.696	2.298
Session	0.092	0.315	.772	−0.531	0.753
Trial	0.062	0.119	.584	−0.189	0.295

^†^ *p* < .10; ** *p* < .01

^a^Reference level = Simultaneous I, ^b^ reference level = giant otters

Performance in all conditions was compared to the Simultaneous I condition (see Table 1). There was a tendency for higher success in the Simultaneous II condition. This could be due to learning or higher motivation because the experimenters compensated for the tilting of the board. In the crucial comparison between the Simultaneous I condition and the Delay I condition, we found that success was significantly lower in the Delay I condition. Figure 4 depicts the substantial reduction in coordination success between the conditions in both species. A direct comparison between Delay I and Delay II found no significant increase [average GLMM estimate: *β* = 0.38, *p* = .496, 95% CI (−8.38: 2.99)] suggesting the experience with the longer rope in the Long-rope-delay condition did not improve performance.

Interestingly, there was no significant difference in success between the Simultaneous I and Long-rope-delay condition, suggesting the increase in rope length did reduce the coordination demands, though there was no significant improvement in comparison with Delay I [average GLMM estimate: *β* = 1.74, *p* = .159, 95% CI (−0.90: 4.84)].

In successful trials, giant otters had to wait on average 3.25 s (range: 1–5 s) for a partner to arrive at the board, while small-clawed otters had to wait on average 1.6 s (range: 1–3 s). In unsuccessful trials, the second partner arrived on average after 8.22 s (range: 1–28 s) for giant otters and after 1.83 s (range: 0–13 s) for small-clawed otters. More details about qualitative differences between species can be found in Supplementary Material 1.

## Discussion

In the first comparative experimental study of otter social cognition, individuals of the two otter species spontaneously passed the individual training and solved the cooperative problem-solving task in a well-established paradigm, the loose string task (Hirata and Fuwa 2007). When the two ends of the rope were within reach simultaneously, both giant otter and Asian small-clawed otter pairs were able access the food at high rates. There were no differences in success rates between species, but there were differences across conditions. Otters performed substantially worse as soon as there was a delay between individuals accessing the ropes, requiring the first subject to wait for another one to act together. When we increased the length of the rope to provide an opportunity for subjects to learn the arrival of a partner would lead to success, we found evidence the longer rope did relax synchronization requirements; however, this did not lead to subsequent improvement in performance with the original rope length. Our results do not support the hypothesis that the more socially dependent lifestyle of giant otters would cause higher cooperative problem-solving skills in this experiment. There are several factors that should be taken into consideration; these will be discussed below.

Otters (and other species that do not succeed in the delay task) could either not inhibit pulling the rope or did not understand the task contingencies sufficiently and therefore pulled the rope as soon as they could reach it. This resulted in low success rates in the delay condition. They were, however, successful in the simultaneous conditions. With this result, they are in good company with various species known for their high cognitive skills such as rooks (Seed et al. 2008), African grey parrots (Péron et al. 2011) and ravens (Massen et al. 2015) that were all tested in the loose string task with similar methods and all showed similar results. Importantly, all these species also failed a delay condition. This leads to the question of what the simultaneous condition can tell us about cooperation, when success can be achieved as a by-product of individual actions. This is particularly important considering there are several studies using the loose string task that did not include a delay condition at all (e.g., Hare et al. 2007; Drea and Carter 2009; Scheid and Noë 2010). Succeeding in the simultaneous condition clearly does not suffice to claim complex social cognitive abilities, but it is a successful behavior nonetheless. It could also be argued that successful cooperation in the wild, such as cooperative hunting, may also depend more on situational coordination and by-product mutualism than on cognitive skills and an understanding of a partner’s role (Gilby and Connor 2010). Success in the delay task, however, appears necessary to draw any conclusions about the ability of a species to coordinate their actions for cooperation (according to the definition of Boesch and Boesch 1989).

In the current version of the loose string task, the otters were tested in their social group, increasing the ecological validity of the situation. Given the feeding ecology of giant otters (foraging in groups) versus Asian small-clawed otters (foraging individually), we expected this setup to advantage giant otters. Our results suggest this was not the case. It is possible that the group setting, instead of fostering more natural cooperative behaviors, increased competition which might in turn have promoted faster, less inhibited decision making and thus poor performance in the delay task. Tolerance has previously been found to play an important role in task success (Hare et al. 2007; Schwing et al. 2016), and neither of the two previous species presented with the loose string task in a group passed the delay task (Massen et al. 2015; Molesti and Majolo 2016). We noted a species difference in the composition of pulling pairs: A single pair was responsible for the vast majority of all successful trials in giant otters (Madija and Otto), whereas successful pairs in small-clawed otters were more balanced across individuals, suggesting the level of tolerance in small-clawed otters is higher. However, the group composition differed between groups: Both groups were made up of family members, as is typical for both species, but in the giant otters the adult male had died the previous year, and in the small-clawed otters, there was no breeding pair, only siblings. It is difficult to predict how these differences may have affected the behavior in the test. Future research might aim at testing otters in a more controlled setting to look at success rates of dyads and to investigate the effect of the group setting. Unfortunately, to avoid major disruption of group cohesion it was not possible for us to separate the giant otters.

The current study is the first comparative social cognitive study conducted with otters. It is therefore a first step to explore the socio-cognitive capacities of these species known for traits suggested to be an indication of complex cognitive skills in other taxa, e.g., cooperative breeding and hunting, large relative neocortex size (compared to other carnivores; Dunbar and Bever 1998), neophilia and social complexity (Byrne and Whiten 1988; Humphrey 1976). We have a clear-cut result: Both giant otters and Asian small-clawed otters succeeded in solving the social problem of our version of the loose string task when pairs could reach the ends of the rope simultaneously. In both species, this success broke down as soon as a delay was introduced. Otters’ failure to wait for a partner suggests that they either did not understand the task contingencies or could not inhibit pulling a rope as soon as it was available. This initial finding should be explored in more detail in the future.

## Electronic supplementary material

Below is the link to the electronic supplementary material.

Supplementary material 1 (DOCX 22 kb)

Supplementary material 2 (MP4 47473 kb)

Supplementary material 3 (XLSX 30 kb)
