# Supplementary material for: Cooperative problem solving in giant otters (Pteronura brasiliensis) and Asian small-clawed otters (Aonyx cinerea)
Source: Anim Cogn. 2017 Aug 24;20(6):1107–14. doi: 10.1007/s10071-017-1126-2 (PMC5640742; doi:10.1007/s10071-017-1126-2)
Supplement: Supplementary file 1 — Supplementary material 1 (DOCX 22 kb) [file 10071_2017_1126_MOESM1_ESM.docx]

Title: Cooperative problem solving in giant otters (*Pteronura brasiliensis*) and Asian small-clawed otters (*Aonyx cinerea*)

Journal: Animal Cognition

Authors: Martin Schmelz^1,2,3^; Shona Duguid^1,3^*; Manuel Bohn^1,3^;Christoph J. Völter^1,3,4^

^1^ Department of Developmental and Comparative Psychology, Max Planck Institute for Evolutionary Anthropology, Germany,

^2^ Department of Cognitive Biology, University of Vienna, Austria

^3^ The Otter Project, Leipzig, Germany

^4^School of Psychology and Neuroscience, St Andrews University, UK

*Corresponding Author: [shona_duguid@eva.mpg.de](mailto:shona_duguid@eva.mpg.de); Tel.: +49 (0) 341 3550 431; ORCID iD: 0000-0003-4844-0673

Supplementary Materials

ADDITIONAL METHODS

*Subjects*

Both groups spent the day in spacious indoor enclosures (giant otters: 840 m², small-clawed otters: 130 m²), including large freshwater pool areas (giant otters: 240 m², small clawed otters: 30 m²), and natural vegetation. Small-clawed otters were fed fresh fish, fruits, and vegetables five times a day. The giant otters were provided with fresh fruits, vegetables, eggs, and meat / fish distributed four times a day. The food rewards used for testing replaced their first feeding of the day If individuals did not participate in test trials they always received food at the end of a test session. Subjects could participate in this study voluntarily and were all highly motivated to do so, except for one juvenile giant otter (Erna). During the training, she stopped participating completely and was therefore not included in any analysis, though she was present for all test sessions.

*Procedure*

Details of the training procedure: We used two training platforms simultaneously to reduce potential competition over the food rewards. To access the food on the platform, subjects had to pull the ropes attached to the baited platforms. At the beginning of the training, the platforms were close to the mesh of the enclosure, just outside their reach. Depending on their dexterity we moved the platform further away throughout the training until the platform and the food was at a distance from the mesh comparable to the cooperation test situation. Training continued until every individual had successfully pulled the rope and retrieved the food more than twice within one session. This was achieved by the small-clawed otters in two sessions. After 12 sessions one of the juvenile giant otters (see above) had not reached this criterion, at this point we decided to continue to the test sessions.

*Coding and analysis*

Including the random effects for individual and dyad was necessary because we did not constrain who would participate in a given trial. Including these random effects accounted for the unequal participation statistically and therefore cautioned against interpreting differences on a lower level (dyad composition) might have falsely been attributed to a higher level (differences between species). We also included all possible random slopes within each subject id.

In unsuccessful trials in which only one individual pulled there was no second individual of the dyad. To deal with this in the model, we randomly selected one of the remaining individuals from the same group as the second individual for each of these trials. Please note that in successful trials both members of the dyad could be identified, so that randomly selecting the second individual was only necessary in unsuccessful trials. We repeated this random sampling procedure 1000 times and averaged the corresponding coefficients. Importantly, the random sampling process did not change the number of successful and unsuccessful trials in each condition. All that changed between iterations was the identity of the second individual of the dyad in unsuccessful trials. The values reported in the results section in the manuscript are the average coefficients for each model and the model comparisons. The corresponding data can be found in the Supplementary Material 3. The R-code will be made available upon request.

Furthermore, we coded the identities of the individual that ate the food as well as the one that pulled the rope. In 5.8 % of trials for the giant otters and 23.1% for the small-clawed otters the individual who first pulled the rope was not the individual who ate the food at the end of the trial. These findings highlight potentially interesting species differences. This, in addition to the more balanced participation in the task, suggests that the small-clawed otters may be more tolerant around food than the giant otters.

ADDITIONAL RESULTS

While there were no species differences in performance levels, there were indications of qualitative differences in how this task was solved. Due to the group setting, subjects participated in an unequal number of trials. Table S1 shows how many times each subject participated in each condition. In the giant otter group, participation in the delay conditions (i.e. the "open" conditions in which the ends of the rope were not directly given to the subjects by the experimenters) was very skewed towards two individuals, thus this particular dyad accounted for the majority of successful trials (Madija and Otto; 94% of all successful trials). In the small-clawed otter group, participation in the delay conditions was more balanced (most frequent dyad: Blacky and James; 54% of all successful trials).

**Table S1** *Number of trials in which a subject pulled one end of the rope per condition*

|  | | Condition | | | | |
| --- | --- | --- | --- | --- | --- | --- |
| Subject | | Simultaneous I  (*N^1^*=103) | Simultaneous II  (*N*=93) | Delay I  (*N*=28) | Long-rope-delay  (*N*=29) | Delay II  (*N*=14) |
| Giant otters | |  |  |  |  |  |
|  | Emil | 23 | 21 | 1 | 1 | 0 |
|  | Madija | 100 | 92 | 23 | 29 | 6 |
|  | Otto | 68 | 68 | 9 | 22 | 12 |
|  | Paul | 10 | 5 | 0 | 0 | 0 |
| Small-clawed otters | | |  |  |  |  |
|  | Blacky | 7 | 16 | 15 | 19 | 8 |
|  | James | 75 | 68 | 3 | 10 | 4 |
|  | Rudi | 49 | 45 | 2 | 7 | 3 |
|  | Twoface | 48 | 41 | 11 | 6 | 2 |

^1^Number of trials in each condition
